# XGBoost regression for robust acoustic impedance prediction in the absence of density and sonic logs

**DOI:** 10.1038/s41598-025-24727-9

**Published:** 2025-11-18

**Authors:** Khaled Saleh, Muhammad A. El Hameedy, Walid M. Mabrouk, Ahmed Metwally

**Affiliations:** 1 Department of Geophysics, Faculty of Science, Cairo university, Giza, 12613 Egypt; 2 PetroShahd Company, Zahraa Maadi, Cairo, Egypt

**Keywords:** Acoustic impedance, Machine learning, XGBoost, Seismic inversion, Reservoir characterization, Geology, Geophysics

## Abstract

Acoustic impedance (*Z*) is a fundamental parameter in geophysical subsurface characterization, governing seismic reflection coefficients and enabling reservoir property quantification through seismic inversion. Conventional derivation of *Z* relies on density (*ρ*) and P-wave velocity (*V*_*p*_) logs, yet these datasets are frequently unavailable due to operational constraints, tool limitations, or borehole irregularities. Existing empirical methods, such as neutron porosity-based formulas, suffer from restrictive assumptions -including matrix/fluid constant dependencies, low shale tolerance (< 25%), and negligible secondary porosity - that limit applicability in heterogeneous formations. To overcome these challenges, we present a robust machine learning workflow that predicts Z directly from commonly available well logs, circumventing the need for sonic or density data. A multi-well dataset comprising gamma-ray (*GR*), neutron porosity (*NPHI*), deep resistivity (*R*_*D*_), and formation tops were analyzed. Pearson correlation identified *GR*, *NPHI*, and log-transformed resistivity (*R*_*Dlog*_) as optimal predictors. Data preprocessing included Isolation Forest-based outlier removal and logarithmic resistivity transformation. The XGBoost regressor - selected for its scalability in handling nonlinear interactions - was trained on 80% of the data, with hyperparameters optimized via cross-validated grid search. Model performance was evaluated using mean absolute error (*MAE*), root MSE (*RMSE*), and coefficient of determination (*R²*). The optimized model achieved an R² of 0.916 (training) and 0.808 (testing), with *RMSE* values of 718.3 and 1070, respectively. Independent validation on a blind well demonstrated strong generalization (*R²* = 0.869, *RMSE* = 981.3), with predicted *Z* logs showing stratigraphic fidelity and suppression of high-amplitude artifacts inherent to sonic-derived impedance. Compared to empirical methods, the ML workflow eliminates reliance on matrix/fluid constants, accommodates shale volumes > 25%, and mitigates errors from secondary porosity or gas effects. This provides a scalable, cost-effective solution to enhance seismic inversion accuracy in data-scarce or complex lithological settings.

## Introduction

### Background

Acoustic impedance (*AI* or *Z*, typically in (g/cm³)·(m/s)) is a fundamental rock property that links borehole logs to seismic data. It is defined as the product of rock density (*ρ*) and P-wave velocity (*V*_p_). Importantly, *Z* correlates inversely with porosity: higher-porosity sandstones tend to have lower acoustic impedance than tighter shales^1^. This makes *Z* a key seismic attribute for reservoir analysis - low-*Z* zones often indicate porous, hydrocarbon-bearing sands, while high-*Z* zones suggest compact shale or carbonates^2^. Quantitative seismic inversion routinely targets *Z* because it provides a continuous subsurface impedance volume that can be directly related to lithology and fluid content^3^. For example, it has long been recognized that multi-attribute inversion of seismic to *Z* can greatly reduce uncertainty in reservoir forecasting^4,5^ and low *Z* anomalies often guide well placement in heterogenous fields. In short, accurate *Z* logs or volumes are invaluable for seismic inversion, amplitude-versus-offset (*AVO*) analysis, porosity estimation, and overall reservoir characterization^6–8^.

Conventionally, acoustic impedance is computed directly from well logs using the relationship Z = ρ · V_p_. Here, *V*_p_ is obtained from a sonic (transit-time) log and *ρ* from a bulk-density log (*RHOB*). When both logs are available, *Z* can be calculated straightforwardly. However, in many wells (especially older or frontier wells) one or both logs may be missing. In such cases, petrophysicists resort to empirical or rock-physics approximations. For example, Gardner’s Equation^9^. and related velocity–density trends can estimate ρ from V_p_, while Castagna mudrock relations^10^ or Wyllie time-average models can estimate *V*_p_ from porosity or other logs^11^. These methods require assumptions about lithology (e.g. pure quartz vs. calcite matrix), shale content, and fluid type. Alternatively, inversion workflows use well logs to build a low-frequency model for seismic inversion, but this again relies on crude velocity - depth curves or assumed matrix velocities. In practice, these approaches can introduce significant errors: empirical transforms may fail in complex sedimentary facies or poorly understood lithologies, and inversion without a calibrated low-frequency trend tends to bias Z values^12,13^.

Furthermore, Mabrouk and Hassan proposed a petrophysical inversion formula to estimate acoustic impedance directly from neutron porosity logs when both sonic and density data are absent or unreliable^14^. Their approach combines the density response equation, elastic impedance definition, and Raiga-Clemenceau et al. transit-time model to derive Z as a function of neutron-derived porosity, fluid and matrix densities, and matrix-specific exponent^15^. Applied to Gulf of Suez Basin wells, it achieved correlations exceeding 0.93 in clean formations and ≤ 10% error when shale volume remained below 25%, though accuracy diminished in shaly intervals (> 25% Vsh) due to bound water effects. While effective in data-poor wells and rough-hole conditions, this method still relies on assumptions of constant fluid/matrix properties and no secondary porosity.

To overcome these limitations, literature has explored both empirical correlations and machine-learning (ML) models to estimate acoustic impedance or infer missing logs. Some studies use seismic attributes or other logs as proxies. For instance, Surachman et al. used nine post-stack seismic attributes (plus depth and two-way travel time) as inputs to supervised ML regressors (Random Forest, Extra Trees, Neural Network) to predict well-log Z. They achieved an *R*² ≈0.85 for the neural network model, with ensemble trees (Extra Trees) yielding lower error^16^.

Other work targets direct log predictions. For example, Khan et al. used XGBoost and neural networks to predict missing logs in a fluvio-deltaic reservoir; their model achieved *R*²≈0.87 for Z on test wells^17^. In petrophysics, Garini et al. introduced a workflow to handle incomplete well-log datasets for lithology classification. They compared XGBoost, KNN, and ANN for imputing logs and found that XGBoost delivered the lowest error for density (*RHOB*) and neutron-porosity (*NPHI*) logs in wells with up to 30% missing data^18^.

Deep learning approaches have also been applied to log imputation. For instance, Al-Fakih et al. developed sequential generative models to generate synthetic well-log sequences and impute gaps. Their bidirectional recurrent network effectively exploits depth-wise correlations in logs^19^. Other authors have used convolutional or LSTM networks to predict sonic or density from gamma-ray, resistivity, and porosity curves^20–22^. These methods demonstrate that complex nonlinear relationships among logs can be learned from data, but most focus on recovering missing intermediate logs (like sonic) rather than directly outputting impedance. Recent studies have applied machine learning and deep learning methods to reservoir property estimation. For example, deep convolutional neural networks have been used to predict elastic properties (Vp, Vs, and density) from seismic data, achieving improved lateral continuity compared to conventional inversion approaches^23^. Similarly, workflows integrating synthetic training data with random forest regression have been developed to reduce facies-related bias in ML-based acoustic impedance inversion, demonstrating improved prediction accuracy and facies imaging in both synthetic models and field applications^24^.

### Research gap and roadmap

Despite these advances, a key gap remains direct prediction of acoustic impedance from only commonly available logs (e.g. gamma-ray, neutron-porosity, deep resistivity) without using sonic or density curves. Most ML studies either perform a form of inversion or predict one log from another (e.g. sonic from *GR*/*NPHI*). Very few attempt to learn the mapping to Z itself from standard petrophysical logs. Critically, our approach avoids explicit rock-physics assumptions about shale volume or porosity that underlie empirical methods. For instance, classical density and velocity estimation often requires assumed matrix and fluid properties; by contrast, an ML model can implicitly learn these relationships from multi-well data. This study is novel in using XGBoost to directly predict Z from *GR*, *NPHI*, and resistivity logs filling the gap between missing-log imputation and inversion: we bypass intermediate sonic/density estimation entirely. In doing so, we provide a new tool for petrophysicists to generate Z logs where only basic logs exist.

This work has important implications for frontier exploration and data-poor reservoirs. In early-stage fields, often only gamma, porosity, and resistivity logs are available (core or sonic surveys may be missing or prohibitively expensive). Yet acoustic impedance is needed for tying wells to seismic. By leveraging machine learning, our method can produce synthetic Z logs to aid inversion and reservoir modeling. Improving Z estimates under log constraints can reduce uncertainty in reservoir characterization and accelerate decisions in “exploration vintages” with sparse data. The adopted approach XGBoost-based Z prediction directly addresses the need for robust petrophysical estimation in wells where traditional data are incomplete, offering a path toward more reliable seismic inversion and reservoir Characterization in challenging settings.

## Methodology

The study follows a structured machine learning workflow, beginning with data collection and preprocessing. Preprocessing steps include outlier removal and feature engineering, alongside the computation of the target acoustic impedance using a basic empirical equation. To enhance feature-target relationships, the deep resistivity log is log-transformed, improving its correlation with the target. Subsequent stages involve exploratory data analysis and selection of the most relevant input features. The dataset is then partitioned into training and testing subsets for model development and hyperparameter optimization. Additionally, an independent blind well is reserved for evaluating the model’s generalization and practical applicability.

**Fig. 1:**
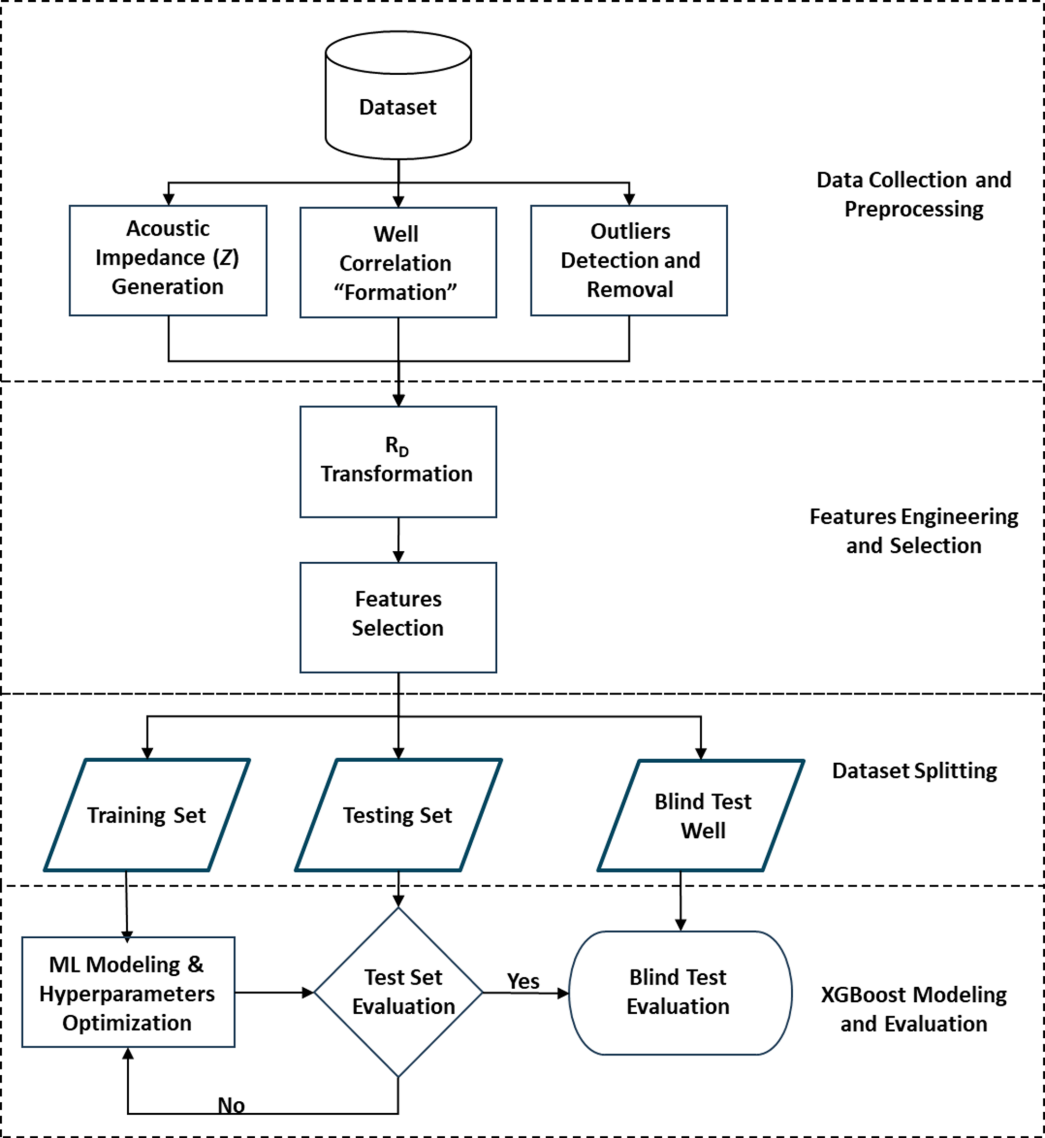
Machine Learning Workflow for acoustic impedance prediction.

Although workflow diagrams for machine learning often appear generic, Fig. 1 is included to highlights how the standard ML pipeline is adapted to the geoscience domain. Specifically, it emphasizes three critical domain-driven steps: (i) acoustic impedance generation as an input feature derived from seismic data, (ii) well correlation across formations to ensure stratigraphic consistency, and (iii) blind well evaluation as a validation approach that mimics real-world scenarios where models are applied to entirely unseen wells. These steps are not part of generic ML workflows but are essential when integrating machine learning with subsurface data. The figure therefore provides readers with a clear and concise visual representation of how conventional ML methods are specialized for petroleum geoscience applications.

### Data collection and preprocessing

The dataset comprises well log data from six boreholes drilled in the Shahd SE Field, located in the northern part of Egypt’s Western Desert. Specifically gamma-ray (*GR*), neutron porosity (*NPHI*), and deep resistivity (*R*_*D*_) logs were used for this study. The gamma-ray log records natural radioactivity (from K, U, Th) of the formation and is commonly used to infer shale content (high GR values typically indicate clay-rich layers). The neutron porosity log measures hydrogen content and thus serves as a proxy for fluid-filled porosity in the formation. The deep resistivity log measures the formation electrical resistivity (Ω m), which distinguishes hydrocarbon-bearing (high resistivity) from water-bearing (low resistivity) zones. These three logs are the *input features* for machine learning; sonic (*DTC*) and density (*RHOB*) logs are not available as predictors.

#### Acoustic impedance generation

The acoustic impedance is the *target* variable for prediction. acoustic impedance is defined as the product of bulk density *ρ* and compressional wave velocity *Vp*​:

1 $${\text{Z }} = \rho \cdot Vp$$

When available, compressional slowness (sonic log *DTC*, in µs/ft) and formation density (*RHOB*, in g/cm³) were used to compute *Vp* ​ and hence Z. Assuming DTC is in microseconds per foot, the compressional velocity *Vp* (in ft/s) is computed as *Vp* = 10^6^. 0.3048 / DTC. Converting units if necessary (e.g. to m/s) can be performed consistently. The computed velocity is then multiplied by the density (converted to kg/m³) to yield impedance in SI units.

#### Well correlation

**Fig. 2:**
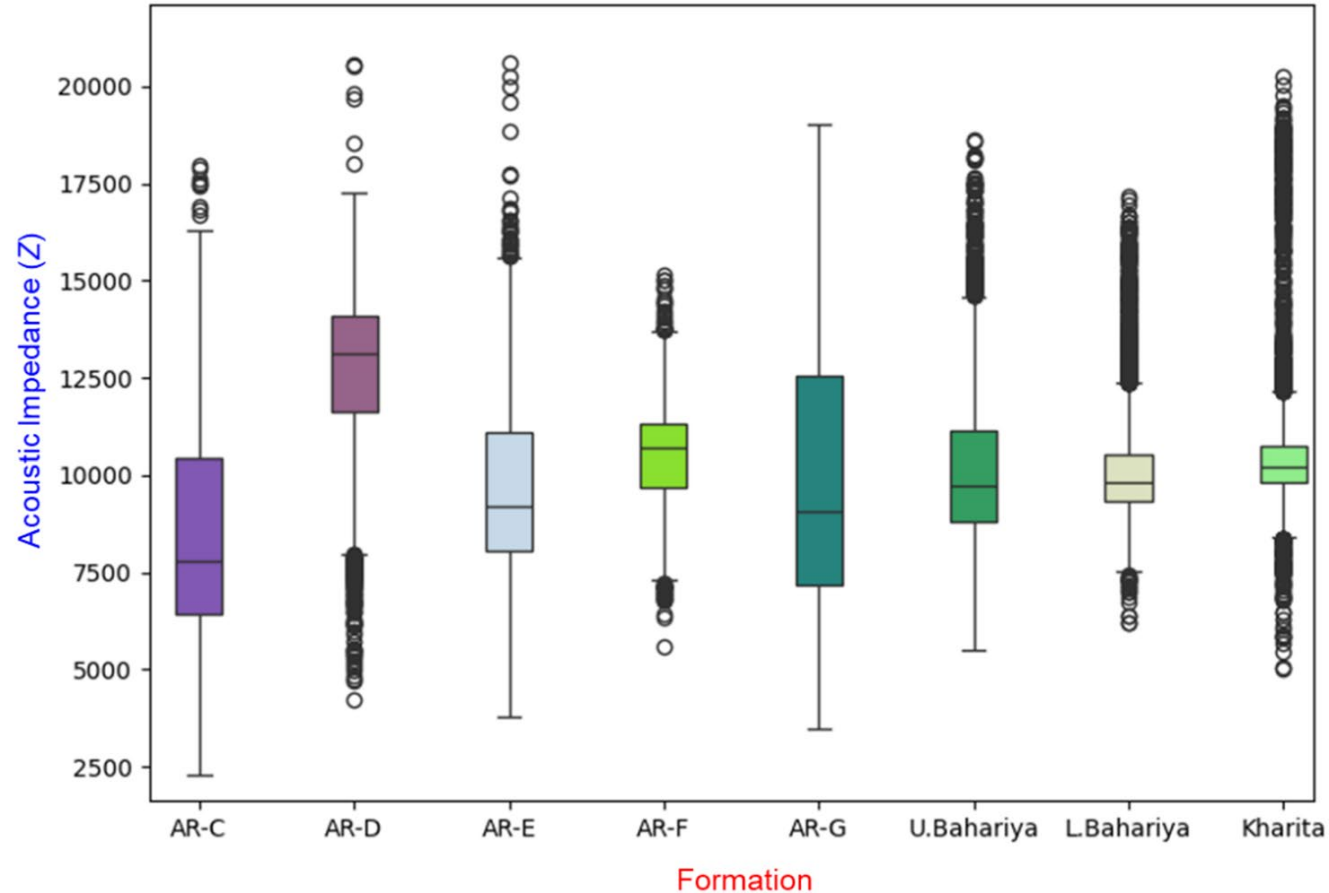
Boxplot illustrating the distribution of acoustic impedance (Z) values across different geological formations.

Incorporating a formation variable as an additional input feature for acoustic impedance modeling significantly enhances the predictive performance and geological consistency of the model. This variable, interpreted from conventional E-logs (such as GR, Resistivity and NPHI logs), serves as a proxy for the stratigraphic framework and lithological variations within the subsurface. By introducing formation as a categorical or encoded variable, the model can better distinguish between the distinct petrophysical behaviors of different formations. Each formation typically exhibits a limited and characteristic range of acoustic impedance values due to differences in mineralogy, porosity, and compaction (Fig. 2). As a result, the formation variable provides crucial contextual information that constrains the model’s predictions, reduces uncertainty, and minimizes the overlap in impedance values between geologically unrelated units. This leads to improved generalization and a more geologically meaningful prediction of acoustic impedance across heterogeneous intervals.

Categorical features such as formation were transformed using OneHotEncoding, which converts each formation into a binary indicator without imposing artificial numeric order on stratigraphic units. This allowed the model to capture formation-specific patterns in the log-to-impedance mapping, improving the consistency and stability of predictions.

#### Outlier detection and removal

**Fig. 3:**
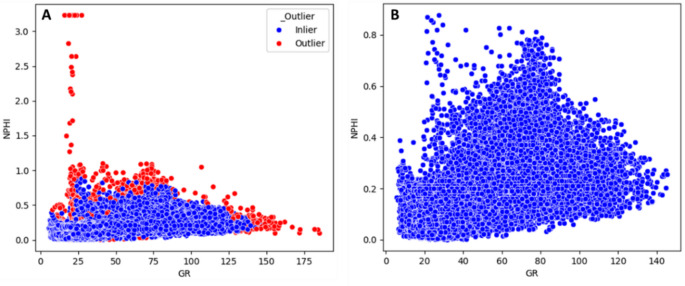
Gamma Ray log (GR) versus Neutron log (NPHI) cross-plot (**A**) outliers detection (red points) and (**B**) after removing outliers.

Outlier detection is then performed on the log features (Fig. 3) to remove spurious measurements (e.g. tool noise or borehole effects). We use the Isolation Forest algorithm for anomaly detection. Isolation Forest is an unsupervised tree-based method that isolates outliers by randomly partitioning data: anomalies require fewer random splits to isolate because they are “few and different” In practice, we train an Isolation Forest on the multi-log feature set. Points flagged as anomalies (typically based on an isolation score threshold or contamination parameter) are excluded from further analysis. This process ensures that extreme values do not unduly bias the model training.

Removing these anomalous points serves as a data conditioning step rather than an optimization variable: retaining them would bias the learning process, distort the log-to-log relationships, and reduce the generalizability of the model. The subsequent XGBoost model is therefore trained and evaluated exclusively on the cleaned dataset, ensuring that predictive performance reflects geologically meaningful trends rather than spurious noise.

### Features engineering and selection

#### Data transformation

Prior to modeling, raw log curves are cleaned and standardized. In particular, the deep resistivity values span several orders of magnitude, so we apply a logarithmic transform to compress the dynamic range. Specifically, we compute:

2 $$R_{{D\log }} = {\text{log}}_{{{\text{1}}0}} \left( {R_{D} } \right)$$

**Fig. 4:**
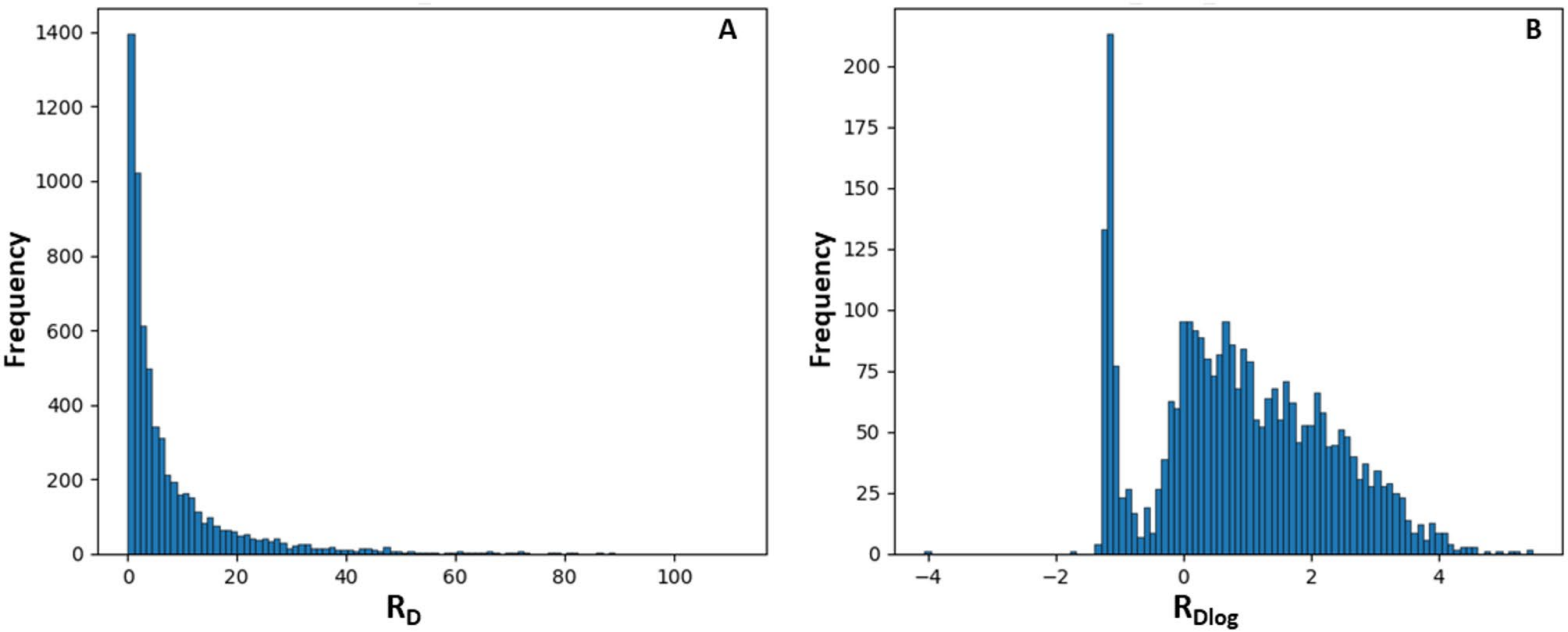
Histogram distributions of the deep resistivity (RD) log. (**A**) Original RD values showing a highly right-skewed distribution. (**B**) Log-transformed RD values, exhibiting reduced skewness and a more normalized distribution.

Where *R*_*D*_ is resistivity in Ω m. Figure 4 presents the histogram distributions of the original *R*_*D*_ log and its log-transformed counterpart. The transformation effectively reduces skewness and enhances linearity, thereby improving correlation with other input logs and the target acoustic impedance^22^.

#### Feature selection

To identify the most predictive inputs, we compute the Pearson correlation coefficient (r) between each candidate log feature and the target impedance *Z*. The Pearson r measures linear association and is defined as:

3 $$\:r=\:\frac{\sum\:\left({x}_{i}-\overline{x}\right)\left({y}_{i}-\overline{y}\right)}{\sqrt{\sum\:{\left({x}_{i}-\:\overline{x}\right)}^{2}.\:\sum\:{\left({y}_{i}-\overline{y}\right)}^{2}}}\:$$

Where $\:{x}_{i}$ and $\:{y}_{i}$ are the data points of variables $\:X$ and $\:Y$ repectively, $\:\overline{x}$ is the mean of $\:X$ and $\:\overline{y}$ is the mean of $\:Y$.The coefficient r ranges from − 1 to + 1, with values near ± 1 indicating strong linear correlation.

To complement traditional correlation-based feature selection, SHapley Additive exPlanations (SHAP) were employed as an additional method to assess feature importance for the XGBoost model. SHAP is an approach that attributes the contribution of each feature by computing its marginal effect on the model output, averaged across all possible feature coalitions. Unlike correlation analysis, which captures only linear relationships, SHAP accounts for nonlinear dependencies and feature interactions, providing a consistent and interpretable measure of feature relevance. This makes it particularly suitable for complex machine learning models such as XGBoost.

### Dataset splitting

The data from the six wells were partitioned as follows: five wells were used for model development, with 80% of their data allocated for training and 20% reserved for testing to optimize model performance. The sixth well was held out entirely as a blind test well, serving as an independent dataset for the final evaluation of the model’s predictive capability.

### Modeling training and evaluation

In this study, the eXtreme Gradient Boosting (XGBoost) algorithm was adopted as the predictive model. XGBoost is an ensemble method that builds upon decision trees by applying gradient boosting, where successive trees are trained to correct the residual errors of previous ones. This approach provides several advantages that are particularly relevant for subsurface data. First, it improves predictive accuracy and generalization compared to a single decision tree, which, while effective, can be sensitive to data variability. Second, XGBoost incorporates built-in regularization (L1 and L2) that mitigates overfitting, a common risk when dealing with noisy and heterogeneous well log and seismic datasets. Third, XGBoost naturally captures nonlinear relationships and complex feature interactions, which are typical in geological systems. Finally, it allows feature importance analysis, supporting interpretability of the model results. These characteristics make XGBoost a suitable and robust choice for well log prediction tasks, while maintaining the interpretability of tree-based approaches that have previously shown promising results in similar studies.

#### XGBoost regression model

We implement a gradient-boosted tree ensemble using XGBoost for regression. XGBoost (eXtreme Gradient Boosting) is an optimized implementation of gradient-boosted decision trees that delivers high predictive performance on tabular data. In this framework, the model prediction $\:{\widehat{y}}_{i}$ for sample $\:i$ is the sum of outputs from K regression trees:

4 $$\:{\widehat{y}}_{i}=\:\sum\:_{k=1}^{K}{f}_{k}\left({x}_{i}\right)\:$$

Where each $\:{f}_{k}$ is a tree-valued function mapping features $\:{x}_{i}$ to a real-valued score. Learning proceeds *additively*: at each boosting step $\:t$, we add a new tree $\:{f}_{t}$ to improve the current predictions $\:{{\widehat{y}}_{i}}^{(t-1)}\text{}$​. The updated prediction is

5 $$\:{{\widehat{y}}_{i}}^{\left(t\right)}=\:{{\widehat{y}}_{i}}^{(t-1)}\:+{f}_{t}\left({x}_{i}\right)$$

With $\:{{\widehat{y}}_{i}}^{\left(0\right)}\:$initialized, often to the mean of the target variable. The boosting process continues for $\:t$ =1,…, K, sequentially reducing the residual errors of the ensemble at each step.

#### Objective function and regularization

The XGBoost algorithm optimizes a regularized objective function at each iteration^25^. For regression with mean-squared-error (MSE) loss, the overall objective after t trees is:

6 $$\:{obj}^{\left(t\right)}=\:\sum\:_{i=1}^{n}{({y}_{i}+\:{{\widehat{y}}_{i}}^{\left(t\right)})}^{2}+\:\sum\:_{k=1}^{t}{\Omega\:}\left({f}_{k}\right)$$

Where $\:{y}_{i}$ are true target values and $\:{\Omega\:}\left({f}_{k}\right)$ is a complexity penalty on tree $\:\text{f}$. In practice, XGBoost uses a second-order approximation of the loss. Expanding the loss via a Taylor series around the previous predictions, we obtain for step $\:\text{t}$:

7 $$\:{obj}^{\left(t\right)}\:\approx\:\:\sum\:_{i=1}^{n}\left({g}_{i}{f}_{t}\left({x}_{i}\right)+\:\frac{1}{2}\:{h}_{i}{f}_{t}^{2}\left({x}_{i}\right)\right)+{\Omega\:}\left({f}_{t}\right)+const$$

Where:

$$\:{g}_{i}=\:\frac{\partial\:L\:({y}_{i},\:{\widehat{y}}_{i})\:}{\partial\:{\widehat{y}}_{i}\:}\:=\:{\widehat{y}}_{i}-\:{y}_{i}\:\:$$

$$\:{h}_{i}=\frac{{\partial\:}^{2}L\:({y}_{i},\:{\widehat{y}}_{i})\:}{\partial\:{{\widehat{y}}_{i}}^{2}\:}=1$$

Here $\:{g}_{i}$ and $\:{h}_{i}\:$represent the first and second derivatives (i.e., gradient and Hessian) of the loss with respect to the prediction. By optimizing this second order (quadratic) approximation, XGBoost efficiently determine the next tree $\:{f}_{t}$ to add to the ensemble.

The regularization term Ω($\:f$) serves to control model complexity to avoid overfitting. Each decision tree $\:f\left(x\right)$ is defined as $\:f\left(x\right)={w}_{q\left(x\right)}$ ​, where $\:q\left(x\right)$ maps an input $\:x$ to one of $\:T$ leaves, and $\:w$_j​_ is the score associated with leaf $\:j$. XGBoost formulates the regularization function as:

8 $$\:{\Omega\:}\left(f\right)\:={\upgamma\:}\text{T}+\frac{1}{2}\:{\uplambda\:}{\sum\:}_{\text{j}=1}^{\text{T}}{w}_{j}^{2}$$

Here $\:\text{T}$ is the number of leaves in the tree, γ is the penalty applied for each additional leaf, and λ controls the L_2_​-norm penalty on leaf weights. Increasing γ or λ results in more conservative models by requiring higher gain thresholds for node splits and by shrinking the leaf weights, respectively.

At each candidate split during tree growing, XGBoost computes the gain in the regularized objective. Given a node with aggregated gradient GGG and Hessian HHH, splitting it into left/right subsets yields gain:

9 $$\:\text{Gain}=\frac{1}{2}\left(\frac{{\text{G}}_{\text{L}}^{2}}{{\text{H}}_{\text{L}}+{\uplambda\:}}+\frac{{\text{G}}_{\text{R}}^{2}}{{\text{H}}_{\text{R}}+{\uplambda\:}}-\frac{{\left({\text{G}}_{\text{L}}+{\text{G}}_{\text{R}}\right)}^{2}}{{\text{H}}_{\text{L}}+{\text{H}}_{\text{R}}+{\uplambda\:}}\right)-{\upgamma\:}$$

Where $\:G$_L_, and $\:{H}_{L}$ represent the sum of gradients and Hessians for the left child, while $\:G$_R_,​ $\:{H}_{R}$​ are the corresponding sums for the right child. A split is performed only if the calculated gain exceeds the threshold γ. This formulation incorporates both first-order $\:G$ and second-order $\:H$ information, along with regularization parameters γ and λ, ensuring both efficiency and control over model complexity.

#### Shrinkage and hyperparameter tuning

To improve generalization, each tree contribution is scaled by a learning rate η (shrinkage). After computing $\:{f}_{t}$, the predictions are updated as:

10 $$\:{\widehat{y}}_{i}^{\left(t\right)}={\widehat{y}}_{i}^{(t-1)}+{{\upeta\:}f}_{t}\left({x}_{i}\right)$$

Where η ∈ (0,1) controls the step size in the boosting process. Smaller values of η reduce the risk of overfitting by making the model learn more gradually.

The hyperparameters of the XGBoost model (e.g. number of trees *K*, max tree depth, learning rate η, and regularization γ, λ) are tuned using cross-validation or grid search. In practice, we use K-fold cross-validation on the training set: for each hyperparameter combination, we train the model on k-1 folds and evaluate predictive performance on the hold-out fold. The combination yielding lowest validation error (e.g. RMSE) is chosen. This procedure ensures robust parameter selection and avoids overfitting.

#### Evaluation metrics

To evaluate the predictive performance of the regression models, three established metrics were employed: Mean Absolute Error (MAE), Root Mean Squared Error (RMSE), and the Coefficient of Determination (*R*^2^). These metrics collectively assess accuracy, error sensitivity, and explanatory power, addressing distinct facets of model performance.

##### Mean absolute error (MAE)

The MAE quantifies the average absolute deviations between predicted ($\:\widehat{{y}_{i}}$) and observed ($\:{y}_{i}$) values, computed as:

11 $$\:\text{M}\text{A}\text{E}=\frac{1}{n}{\sum\:}_{i=1}^{n}\left|{y}_{i}-\:{\widehat{y}}_{i}\right|$$

where *n* denotes the total number of observations. MAE is inherently interpretable and robust to scale, as it expresses errors in the same units as the variable of interest. However, since it assigns equal weight to all residuals, it may underrepresent the influence of extreme outliers compared to Root Mean Squared Error, which penalizes large errors more strongly^26^.

##### Root mean squared error (RMSE)

RMSE penalizes larger errors quadratically, offering sensitivity to outliers, and is defined as:

12 $$\:\text{RMSE}=\sqrt{\frac{1}{n}{\sum\:}_{i=1}^{n}{\left({y}_{i}-{\widehat{y}}_{i}\right)}^{2}}$$

This metric is particularly advantageous in contexts where substantial prediction errors carry disproportionately higher operational or financial risks^27^.

Coefficient of Determination ($\:{\varvec{R}}^{2}$).

$\:{R}^{2}$ evaluates the proportion of variance in the dependent variable explained by the model, expressed as:

13 $$\:{R}^{2}=1-\frac{{\sum\:}_{i=1}^{n}{\left({y}_{i}-\widehat{{y}_{i}}\right)}^{2}}{{\sum\:}_{i=1}^{n}{\left({y}_{i}-\overline{y}\right)}^{2}}$$

where $\:\overline{y}$​ represents the mean of observed values. Values approaching 1 indicate near-complete explanatory capability, while values near 0 suggest the model performs comparably to a naïve mean predictor^28^.

## Results and discussion

### Dataset statistics and feature relationships

**Fig. 5:**
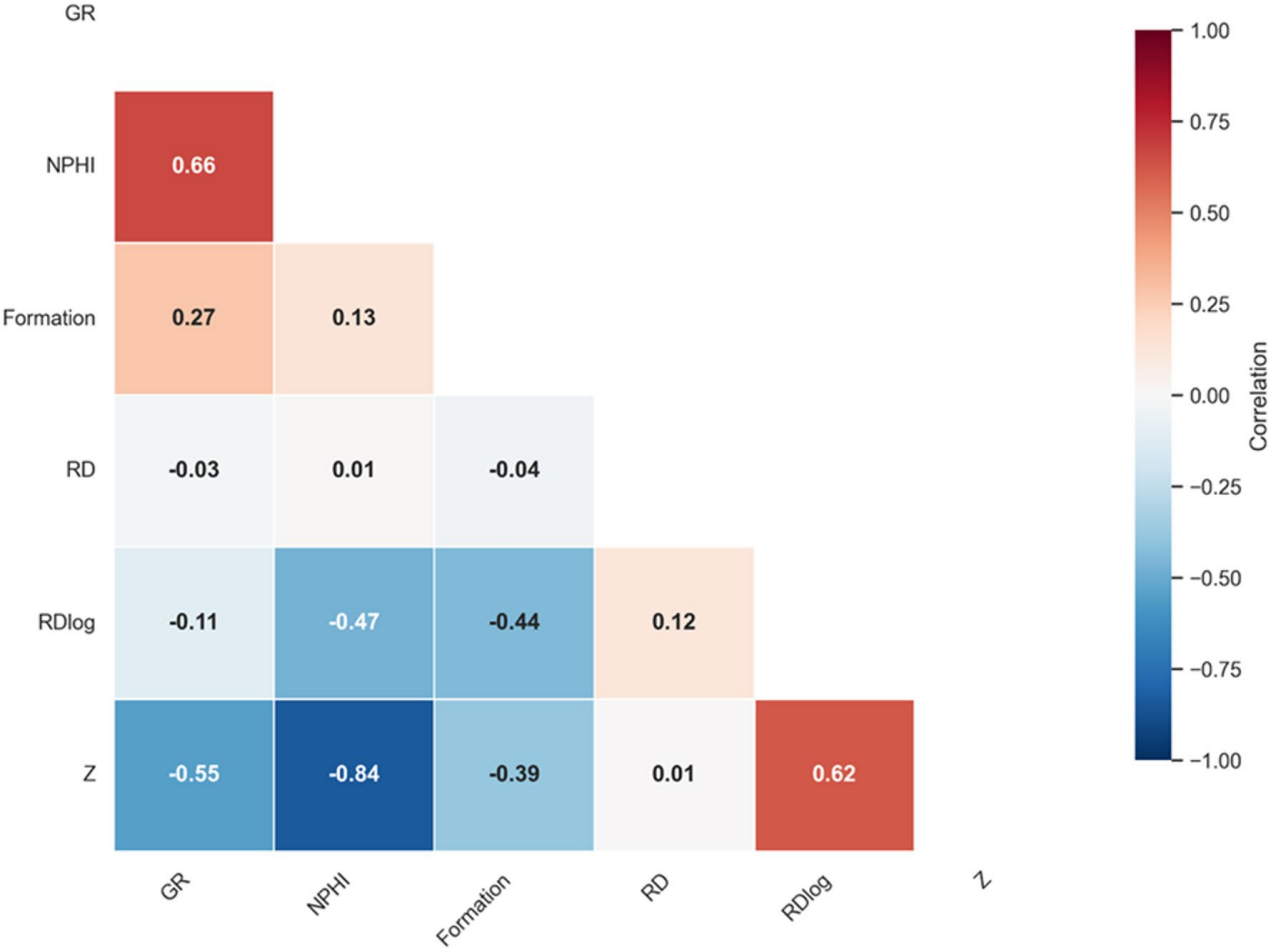
Pearson correlation coefficient for the input feature and the acoustic impedance (*Z*).

Our cleaned dataset comprises sample points from six wells, spanning sandstone, shale, and mixed lithologies. After outlier removal (Isolation Forest flagged ≈ 4% of samples). In our analysis, we computed Pearson correlation (r) coefficients for the input features versus *Z*. Features with higher absolute r are deemed more relevant. Figure 5 presents the Pearson correlation coefficients, indicating that the NPHI log exhibits a strong negative correlation of -0.84 with acoustic impedance. The GR and Formation features show moderate negative correlations of -0.55 and − 0.39, respectively. Although the original RD log demonstrated a very weak correlation (0.01), its logarithmic transformation significantly improved the correlation to 0.62. As a result, the transformed RD log was selected over the original RD log for model training. Table 1 summarize the Pearson correlation coefficients between input logs and target impedance.

**Table 1:** Pearson correlation coefficients between input logs and target impedance.

Feature	Pearson *r* with Z
*R*_*Dlog*_	0.62
*NPHI*	– 0.84
*GR*	– 0.55
*Formation*	– 0.39
*R*_*D*_	0.01

The SHapley Additive exPlanations (SHAP) method was applied to the same cleaned dataset to provide a complementary, model-based perspective on feature relevance. An initial XGBoost model was trained using all candidate features, and SHAP values were computed to quantify each feature’s contribution to predicting acoustic impedance Z. The analysis confirmed that NPHI, R_Dlog_, and GR exert the strongest influence on model predictions, consistent with the Pearson correlation findings, while Formation labels showed a moderate but meaningful contribution. These results (Table 2) support the selection of the same three primary logs, along with Formation labels, as the most informative predictors for subsequent modeling.

**Table 2:** SHAP feature importance ranking from the initial XGBoost model.

Feature	SHAP Importance
*NPHI*	935.08
*GR*	746.28
*R*_*Dlog*_	556.29
*Formation*	335.8
*R*_*D*_	66.4

### Model performance on training and testing sets

The XGBoost regressor was trained on 80% of the data with hyperparameters optimized via 5-fold cross-validated grid search. The best configuration used 500 trees, maximum depth = 6, learning rate η = 0.05, γ = 1, and L_2_ regularization λ = 3.

**Fig. 6:**
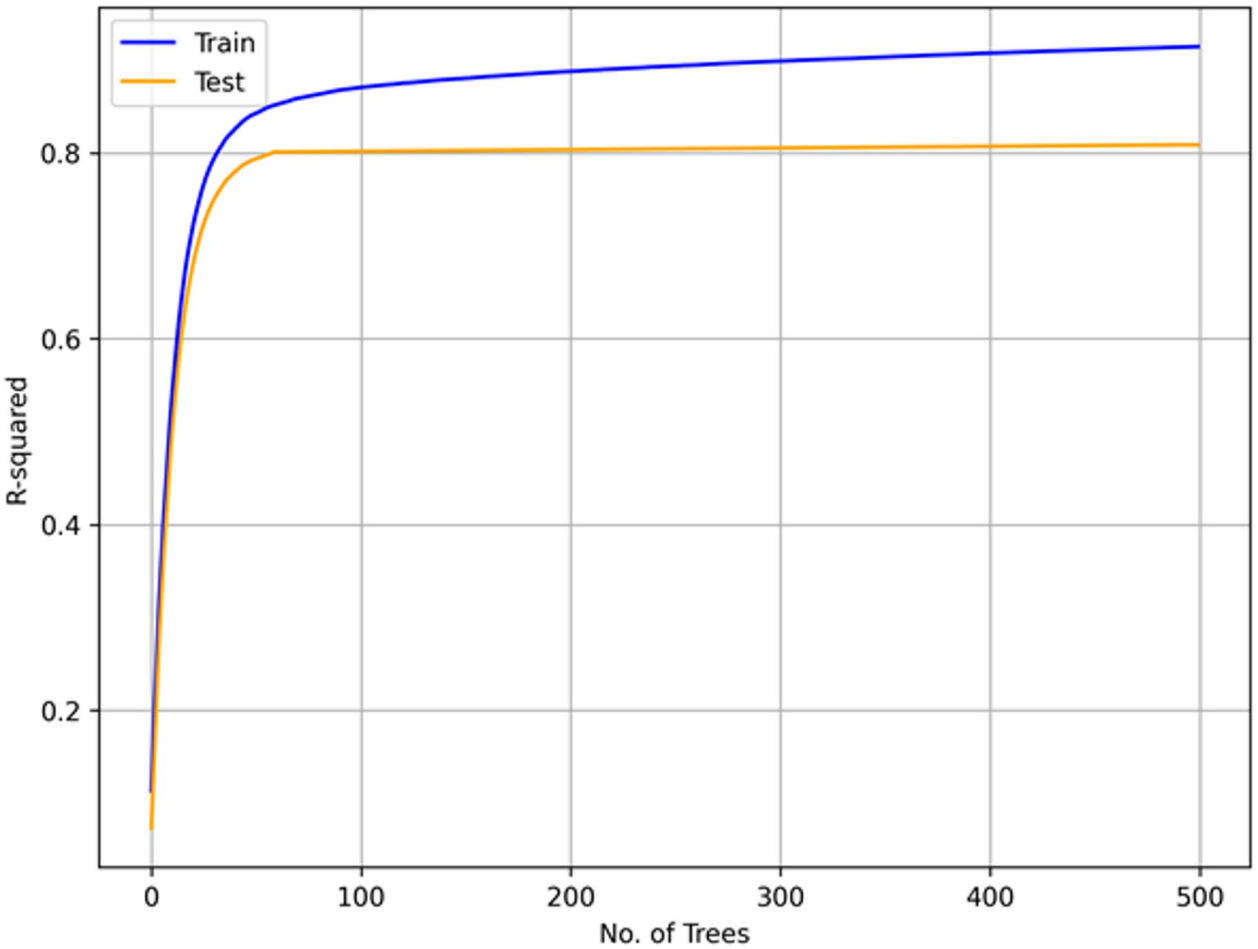
Evolution of model performance with increasing number of trees in the XGBoost model.

Figure 6 shows the relationship between the number of trees (boosting rounds) and the coefficient of determination (R²) for both training and testing datasets. Both curves increase sharply during the early boosting rounds (up to ~ 50 trees), after which the improvement slows down and gradually plateaus. The training R² continues to increase slowly with more trees, while the testing R² stabilizes around 0.80 without significant gain beyond ~ 100 trees. This indicates that the model achieves strong predictive performance early, and additional trees contribute only marginal improvements. The close alignment between training and testing curves demonstrates that the model generalizes well to unseen data, without evidence of underfitting or severe overfitting.

**Fig. 7:**
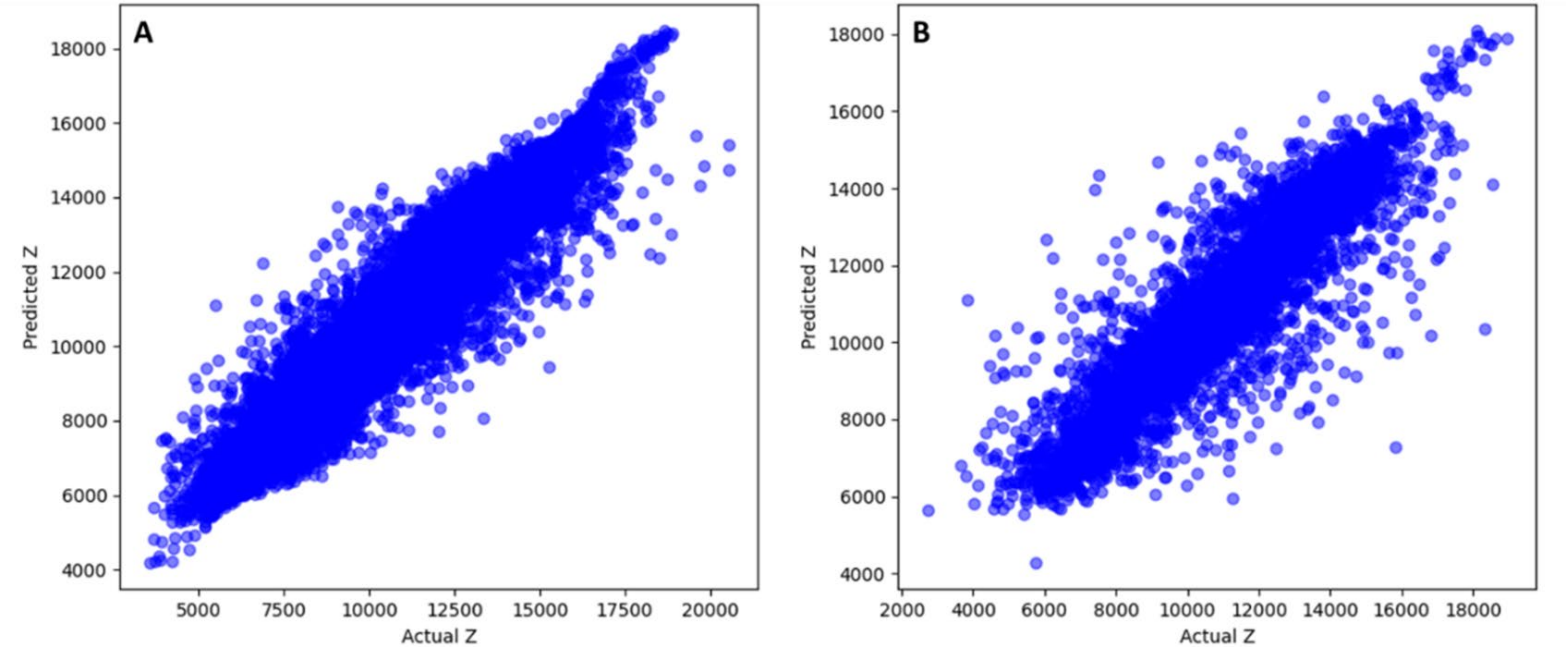
Actual acoustic impedance (Actual$\:\:\text{Z}$) versus predicted acoustic impedance (Predicted $\:\text{Z}$) for (**A**) training set and (**B**) testing set.

The model achieved an R² of 0.916 and RMSE of 718.3 on the training dataset, indicating that over 91% of impedance variance is captured, as shown in Table 3; Fig. 7A. On unseen data, the model retains strong generalization with R² = 0.808 and RMSE = 1070, demonstrating robust learning of nonlinear relationships among logs without overfitting, as presented in Fig. 7B.

**Table 3:** Evaluation metrics for training and testing subsets.

Metric	Training set	Testing set	Blind test
*MAE* (g/cc·ft/s)	480.4	701.6	682.4
*RMSE (*g/cc·ft/s)	718.3	1070	981.3
*R* ^*2*^	0.916	0.808	0.869

**Fig. 8:**
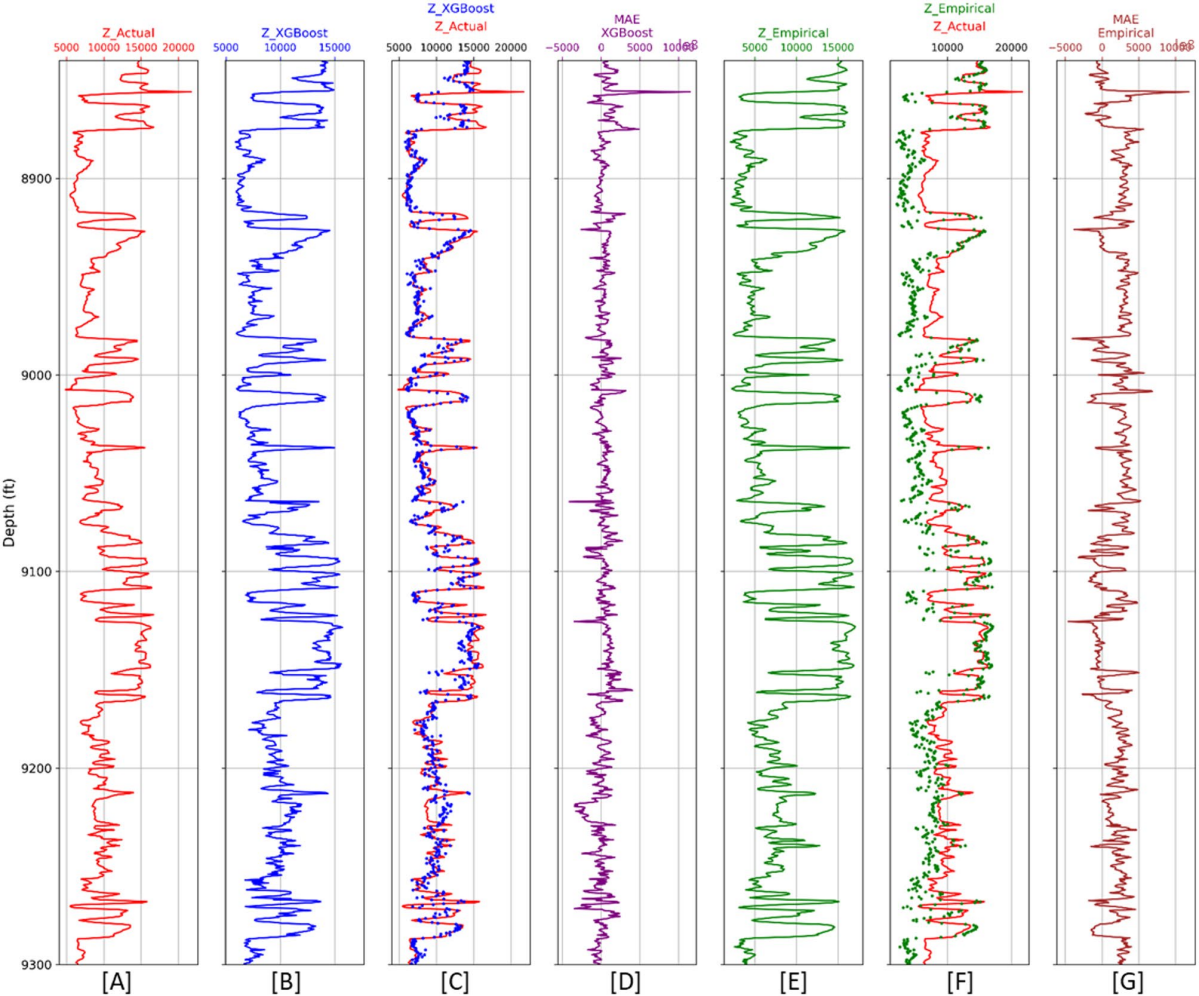
Well-log comparison of acoustic impedance (Z) versus depth. Tracks show (from left to right): actual Z log (**A**), XGBoost-predicted Z log (**B**), overlay of actual and XGBoost predictions (**C**), Mean Absolute Error for XGBoost (**D**), empirical Z from Mabrouk and Hassan’s formula (**E**), overlay of empirical and actual Z logs (**F**), and Mean Absolute Error for the empirical approach (**G**). The visual and error-based comparisons highlight the improved agreement of the XGBoost model with the actual log relative to the empirical method.

To assess model generality, we applied the trained regressor to a blind well withheld entirely from training and testing. In this validation:

- The blind well yielded *R*^*2*^ = 0.869 and *RMSE* = 981.3, closely matching test-set performance and confirming strong spatial generalization as indicated in Table 3.
- Figure 8 shows predicted and actual *Z* logs along the wellbore: the model successfully reproduces stratigraphic impedance trends and attenuates the “spike” artifacts seen in the sonic-derived impedance.

### Comparison with empirical methods

The classical neutron-porosity formula of Mabrouk & Hassan (2013) requires assumed matrix/fluid constants and deteriorates when shale volume exceeds 25% or secondary porosity is present. In our comparative analysis on three wells:

- Empirical approach yielded average errors of 7–10% in shaly intervals and up to 30% in zones with > 25% shale.
- XGBoost model achieved much lower average error across all lithologies, including zones with > 30% shale, without explicit shale-volume thresholds or porosity-type assumptions.

Thus, the ML framework provides good accuracy and broader applicability, eliminating reliance on site-specific matrix/fluid calibrations and empirical saturation limits (Fig. 8).

Our results demonstrate that XGBoost can reliably predict acoustic impedance directly from routinely acquired logs (*GR*,* NPHI*, and *R*_*D*_), filling a critical data gap when sonic or density measurements are missed or of poor quality. This has significant implications for:

1. Frontier exploration: Wells often log only basic curves; our method enables generation of AI logs for seismic tie-in and inversion without additional surveys.
2. Mature fields: Legacy wells lacking sonic/density can now contribute impedance data to updated reservoir models, improving volumetric estimates.
3. Complex lithologies: The model nonlinear learning accommodates shale-rich and mixed facies where empirical transforms fail.

However, limitations include:

- Data dependency: a key limitation of the presented model, common to data-driven approaches, is its dependency on the representativeness of the training data. While the model demonstrates strong generalization within the geological context of the Shahd SE Field, its accuracy may diminish when applied to extreme lithofacies or geological provinces entirely outside the training domain (e.g., evaporites, volcanics, or highly fractured carbonates not encountered in the dataset). This is because the learned relationships between GR, NPHI, RDlog, and acoustic impedance are specific to the system they were trained on. To mitigate this limitation and enhance the model portability, future work will explore the potential of transfer learning techniques. By fine-tuning a pre-trained model on a limited dataset from a new geological domain, it may be possible to efficiently adapt the foundational relationships learned here to novel lithological settings, thereby broadening the applicability of the workflow without requiring extensive retraining from scratch.
- Feature scope: Only three log inputs were used; incorporating additional logs (e.g. shallow resistivity, spectral gamma) could further refine predictions.

Future work may integrate physics-informed constraints or deep learning architecture (e.g. convolutional or recurrent nets) to capture sequence dependencies and further improve generalization to novel lithologies.

## Conclusion

This study successfully established a machine learning framework for the direct prediction of acoustic impedance (*Z*) using commonly available well logs - gamma-ray, neutron porosity, and deep resistivity - effectively bypassing the conventional dependency on sonic and density data. The core achievement lies in the demonstrated capability of the XGBoost algorithm to learn the complex, nonlinear petrophysical relationships governing Z, resulting in a robust model with strong predictive performance.

The primary significance of this workflow is its ability to overcome the fundamental limitations of empirical methods, namely the reliance on assumed matrix and fluid constants and poor performance in shaly or complex lithologies. By providing a reliable method to generate Z logs in data-scarce scenarios, this approach directly enhances the feasibility of seismic inversion and reservoir characterization in frontier exploration and mature field redevelopment.

Future efforts will focus on expanding the model generality across diverse geological domains through transfer learning techniques and integrating a broader suite of input logs to further improve predictive accuracy and operational utility.

## Data Availability

The data supporting the results of this study was obtained from the Egyptian General Petroleum Corporation. Data are however available from the corresponding author upon reasonable and with permission of The Egyptian General Petroleum Corporation.
